# Non-Thermal Dielectric Barrier Discharge (DBD) Effects on Proliferation and Differentiation of Human Fibroblasts Are Primary Mediated by Hydrogen Peroxide

**DOI:** 10.1371/journal.pone.0144968

**Published:** 2015-12-14

**Authors:** Julian Balzer, Kiara Heuer, Erhan Demir, Martin A. Hoffmanns, Sabrina Baldus, Paul C. Fuchs, Peter Awakowicz, Christoph V. Suschek, Christian Opländer

**Affiliations:** 1 Department of Trauma and Hand Surgery, Medical Faculty of the Heinrich-Heine-University, Düsseldorf, Germany; 2 Department of Plastic Surgery, Hand Surgery, Burn Center, Merheim Hospital Cologne, University of Witten/Herdecke, Köln, Germany; 3 Institute for Electrical Engineering and Plasma Technology, Ruhr University, Bochum, Germany; University Paul Sabatier, FRANCE

## Abstract

The proliferation of fibroblasts and myofibroblast differentiation are crucial in wound healing and wound closure. Impaired wound healing is often correlated with chronic bacterial contamination of the wound area. A new promising approach to overcome wound contamination, particularly infection with antibiotic-resistant pathogens, is the topical treatment with non-thermal “cold” atmospheric plasma (CAP). Dielectric barrier discharge (DBD) devices generate CAP containing active and reactive species, which have antibacterial effects but also may affect treated tissue/cells. Moreover, DBD treatment acidifies wound fluids and leads to an accumulation of hydrogen peroxide (H_2_O_2_) and nitric oxide products, such as nitrite and nitrate, in the wound. Thus, in this paper, we addressed the question of whether DBD-induced chemical changes may interfere with wound healing-relevant cell parameters such as viability, proliferation and myofibroblast differentiation of primary human fibroblasts. DBD treatment of 250 μl buffered saline (PBS) led to a treatment time-dependent acidification (pH 6.7; 300 s) and coincidently accumulation of nitrite (~300 μM), nitrate (~1 mM) and H_2_O_2_ (~200 μM). Fibroblast viability was reduced by single DBD treatments (60–300 s; ~77–66%) or exposure to freshly DBD-treated PBS (60–300 s; ~75–55%), accompanied by prolonged proliferation inhibition of the remaining cells. In addition, the total number of myofibroblasts was reduced, whereas in contrast, the myofibroblast frequency was significantly increased 12 days after DBD treatment or exposure to DBD-treated PBS. Control experiments mimicking DBD treatment indicate that plasma-generated H_2_O_2_ was mainly responsible for the decreased proliferation and differentiation, but not for DBD-induced toxicity. In conclusion, apart from antibacterial effects, DBD/CAP may mediate biological processes, for example, wound healing by accumulation of H_2_O_2_. Therefore, a clinical DBD treatment must be well-balanced in order to avoid possible unwanted side effects such as a delayed healing process.

## Introduction

Human skin consists of the two tissue layers, epidermis and dermis. The epidermis is a stratified squamous epithelium composed of proliferating and differentiated keratinocytes.

The underlying thick layer, the dermis, is a collagen-rich connective tissue providing support and nourishment. As the major cell type in the dermis, fibroblasts play a pivotal role in maintaining the skin by synthesis and deposition of various extra cellular matrix (ECM) proteins [1]. Dermal fibroblasts have an important part in the process of wound healing by synthesizing relevant cytokines such as keratinocyte growth factor. In fibroplasia and granulation tissue formation during wound healing, proliferating fibroblasts form a provisional extracellular matrix by generating and depositing collagen and fibronectin [2].

About a week after wounding, a portion of fibroblasts can transform into myofibroblasts, which are primarily known for their key role in wound healing and physiological reconstruction of connective tissue [3]. They express α-smooth muscle actin (α-SMA) and resemble smooth muscle cells in their capacity for generating strong contractile forces [4].

The transformation of fibroblast into myofibroblast begins with the appearance of proto-myofibroblasts expressing beta and gamma cytoplasmic actins containing stress fibers but no alpha-SMA, the marker of differentiated myofibroblasts. This complex process is driven by at least one cytokine, the tissue growth factor-β1 (TGF-β1), extracellular components and mechanical tension [5]. The contractile activity by mature myofibroblasts is beneficial for tissue remodeling and after wound closure, myofibroblasts regularly undergo apoptosis[6]. The persistence of active contractile myofibroblasts can be a detrimental pathological factor in the development of hypertrophic scars, keloids and fibrotic diseases [7,8].

Many factors, such as oxygenation, infection, age, nutrition and diabetes, affect wound healing, leading to improper or impaired tissue repair. The contamination and/or colonization of wounds with bacteria such as *Staphylococcus aureus* (*S*. *aureus*), *Pseudomonas aeruginosa* (*P*. *aeruginosa*), and β-hemolytic *streptococci*, can cause to prolonged wound inflammation driving the wound to a chronic state [9,10]. Often the bacteria in infected wounds occur in the form of biofilms, which are more resistant to conventional antibiotic treatment [11].

A new promising approach to overcome wound contamination, particularly infection with antibiotic-resistant pathogens, is the topical treatment with non-thermal “cold” atmospheric plasma (CAP). In physics, plasma is a fully or partially ionized gas and has been also called the fourth state of matter, after solid, liquid and gas. Plasma can technically be generated by electric gas discharges. By applying strong electric fields, air or other gases can be transformed into the plasma state with the consequent generation of a complex mixture of inter-reacting and short-living ions, atoms, excited species, photons and free electrons [12]. The properties of plasma depend on the type of gas used, gas pressure and discharge geometry.

In medicine, high-temperature plasmas are already used to sterilize equipment as well as to cut and cauterize tissues during surgery [13]. The development of non-thermal plasma devices operating under atmospheric pressure (CAP) makes it possible to treat living matter, for example, to sterilize human skin without thermal damage or obvious adverse effects. It has been shown that non-thermal plasma effectively kills or inactivates a broad spectrum of bacteria [14,15]. Apart from antimicrobial effects, CAP stimulates microcirculation and reveals some antipruritic and anti-inflammatory effects, making the use of CAP attractive in the treatment of many skin diseases [13,16,17].

In general, CAP devices can be categorized into two main types that produce either an “indirect” or a “direct” plasma for treatments [18]. By using a dielectric barrier discharge (DBD) device in dermal application, the human skin represents the counter electrode to ignite the plasma straight onto the body to achieve a direct plasma treatment. Indirect plasma treatment can be obtained by igniting the plasma inside a tube within a flowing process gas such as argon, helium or air. The flowing process gas carries the active particles to the treated object. Such devices are already in use—e.g. plasma torch, plasma jet, plasma needle or plasma pencil. Plasma comprises a mixture of different radical species, UV radiation and a significant flux of charges, which are all potentially influencing biological functions when directly applied to tissue or cells. However, indirect plasma avoids direct contact with the conductive plasma, so no electrical current is drawn by the treated tissue [19,20]. Operated with ambient air, plasma generates reactive nitrogen species such as nitric oxide (NO), nitrogen dioxide (NO_2_) and reactive oxygen species like ozone (O_3_), superoxide (O_2_^-^) and hydroxyl radicals (^-^OH) [21]. In particular, NO is an important molecule that has been shown to regulate many processes in human skin physiology [22,23]. The treatment of liquids and buffers with DBD leads to acidification and accumulation of nitrite and nitrate, which in turn may influence the cell physiology of adult human fibroblasts. In addition, the formation of hydrogen peroxide (H_2_O_2_) has been identified for various plasma sources and settings [24,25]. Apart from its function as an oxidant with antibacterial and cell toxic effects, H_2_O_2_ may serve as a signaling agent in signal transduction pathways in multicellular organisms [26].

The aim of the current study is to evaluate the impact of DBD treatment on viability, proliferation and differentiation of human dermal fibroblasts with focus on the role of DBD- induced nitrite/nitrate/H_2_O_2_ accumulation and acidification.

## Material and Methods

### Materials

If not indicated chemicals were from Sigma (Deisenhofen, Germany).

### Plasma source

The DBD device used has one driven, cylindrical copper electrode covered with aluminum oxide with a total diameter of 10 mm. Plasma was generated by applying voltage pulses with a maximum of ~14 kV and a trigger frequency of 120 Hz.

### Plasma characterization

The plasma was characterized by using absolutely calibrated optical emission spectroscopy (OES). Spectra were measured using a spectrometer (Ocean Optics, QE 65000) with a spectral resolution of about 1.3 nm in the 200–800 nm wavelength range. The averaged gas temperature was determined by comparing the measured rotational temperature of nitrogen at 337.1 nm to a simulated spectrum under the same conditions [27]. Gas temperature (T_g_) was 390 ± 20 K, UV-A was 7.6 ± 2.3 x 10^−3^ mW/cm^2^, UV-B was 1.3 ± 0.4 x10^-3^ mW/cm^2^, electron density was 2.6 ± 0.9 x 10^14^ cm^-3^ and ozone 210 ± 10 ppm. The concentrations and total flux of the generated nitric oxide (NO) and nitrogen oxides (NO_x_) were measured in the gas phase as described previously [28]. The measured amounts of 0.41 ppm NO and 11.42 ppm NO_2_ yields to a flux of Γ_NONO2_ = 5 x 10^18^ cm^-2^ s. At the treatment time of 5 min, the dose of NO/NO_2_ was *Φ*_NONO2_ = 0.75 x 10^21^ cm^-2^, which equals 1.2 mmol.

### Cell culture

Human juvenile foreskin samples were obtained from five patients (5–6 years old), undergoing medically indicated circumcision after written informed consent from parents or legal guardians, with approval of the Ethics Commission of Düsseldorf University (Study No. 3634) and in accordance with the Declaration of Helsinki. Primary cultures of human foreskin fibroblasts were prepared, cultivated and cryoconserved as described elsewhere [29]. For experiments, cryoconserved stocks of fibroblast were thawed and further cultured with cell culture media (DMEM/10% FCS/PEN/STREP) in T 75 flasks (Cellstar, Greiner Bio-One, Frickenhausen, Germany) under normal culture conditions (5% CO_2_, 37°C). For seeding, the cells were detached by two rinses with 0.13 M NaCl and 0.01 M sodium phosphate, pH 7.4 (PBS) and incubated with 0.05% trypsin/0.02% EDTA/0.9% NaCl solution for 3 to 5 min. After the cells became detached, the remaining trypsin activity was neutralized by addition of 10 ml culture media and subsequently centrifuged (5 min/400 x g). After centrifugation, cells were resuspended and counted by using a Neubauer counting chamber and seeded at a cell density of 2.5 x 10^4^ in 24-well-plates (0.79 cm^2^) one day before experiments. All measurements were performed with foreskin fibroblasts in passage 2.

### Plasma treatments of HDF

**Fig 1:**
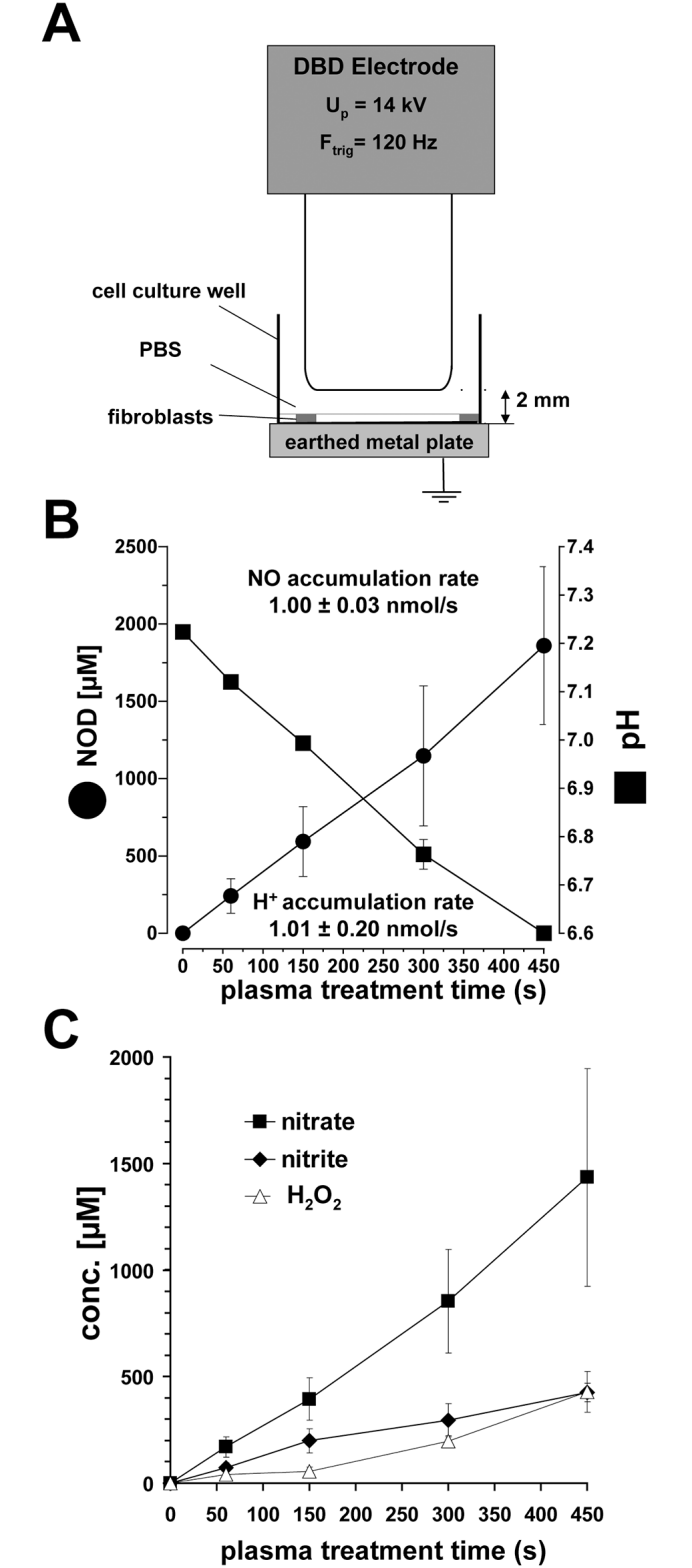
DBD-induced acidification and accumulation of nitrite, nitrate and hydrogen peroxide. **A** Experimental set-up for DBD treatments of fibroblasts. **B** Amounts of NOD (nitrite + nitrate) and pH values obtained after DBD treatment times as indicated. Given are the mean ± sd values (n = 6).

Prior to plasma treatment, cell culture media was carefully replaced with PBS (250 μl) with or without sodium ascorbate (1 mM) after one PBS washing step (500 μl). The cell culture plate was placed on a grounded metal plate under the DBD electrode and the distance between electrode and bottom of the culture plate well was kept as 2 mm (Fig 1A).

For indirect treatments, 250 μl PBS was DBD-treated (0–300 s) and transferred directly after treatment to the cell culture plate with fibroblasts and incubated for 5 min. In addition, for control experiment, fibroblasts were incubated for 5 min with PBS containing varying freshly prepared combinations of H_2_O_2_ (200 μM), nitrite (300 μM), nitrate (1 mM), hydrochloric acid and sodium ascorbate (1 mM). The range of treatment times (60, 150, 300 s) were chosen in accordance with other studies using DBD as plasma source [30,31] and data (not shown) from preliminary experiments with skin fibroblasts. The change of media temperature by DBD treatment (5 min) was less than 0.1°C.

### Determination of pH Values

The pH values of media and buffers were measured using a pH meter (calimatic 766, Knick, Berlin, Germany) and a pH electrode (InLab-micro, Mettler-Toledo, Giessen, Germany). The buffering capacity of PBS was determined by titration with HCl. The obtained data were used to calculate the concentration and accumulation rate of oxonium ions in plasma-treated buffer.

### Detection of nitrite and nitrate

The concentrations of nitrite were quantified by iodine/iodide-based assay NO-analyzer CLD 88 (Ecophysics, Munich, Germany) as described elsewhere [32,33]. To determine the total nitrite and nitrate, samples were added to 0.1 mol/l vanadium (III) chloride in 1 M hydrochloric acid refluxing at 95°C under nitrogen [34]. The calculated NOD amounts were validated by injecting freshly prepared nitrite/nitrate standards into the respective reaction mixtures. Concentrations of nitrate were calculated by subtraction of nitrite from NOD values.

### Toxicity, viability, proliferation and differentiation

For excluding a possible detachment, the cells were observed by a Zeiss light microscope directly after treatment without changing the media in control experiments. Necrotic effects on the cells were investigated by live cell imaging directly after treatment using the fluorescence dyes fluorescein diacetate, Hoechst 33342 and propidium iodide (each dye 0.5 μg/ml). Living and dead cells were analyzed using a fluorescence microscope (Zeiss, Wetzlar, Germany).

Viability of the cells or relative cell numbers in relation to the untreated control were determined by a resazurin-based assay (Celltiterblue, Promega, Madison, WI, USA) at time points as indicated. Briefly, HDF were incubated for 1 h with alamarBlue^™^-reagent (1:20 with Medium; 400 μl) and 2 x 100 μl were taken for direct measurement at room temperature using a fluorescence spectrometer (VICTOR II Plate Reader, PerkinElmer, Waltham, MA, USA) at excitation wavelength 540 nm, emission wavelength 590 nm. The linear range of growth curves were used to calculate doubling times.

On day 12 after treatment, the cells were fixed with paraformaldehyde/PBS (4%; 15 min), washed 3x with PBS and permeabilized by 0.2% Triton X-100/PBS. After incubation with blocking buffer (4% BSA/PBS; 12 h; 4°C), the cells were stained with mouse anti—SMA-antibodies (1:400; 60 min; 37°C). Then the cells were washed 3x with PBS and incubated for 60 min with an AlexaFluor488-conjugated goat-anti-mouse antibody (1:1000; Invitrogen, Carlsbad, CA, USA) in blocking buffer. After three washing steps, the cells were incubated with Hoechst 33342 (1 μg/ml) in PBS for 10 min and visualized by using an invert fluorescence microscope. Photos (4x) of each well were taken and the total number of cells, area of single cell nucleus and the area of -SMA^+^-signal were determined by using Fiji (National Institutes of Health, Bethesda, MD, USA) [35].

### Measurement of H_2_O_2_

The concentration of H_2_O_2_ in DBD-treated PBS was determined by the titanium oxide oxalate method [36]. Briefly, a stock solution of potassium dihydrate (0.1 M) with 2 M sulfuric acid was freshly prepared.

Directly after DBD treatment, the tested solution (50 μl) was taken and diluted 1:4 with PBS, before 200 μl of the titanium oxide oxalate stock solution was added. The reaction of potassium titanium oxide with H_2_O_2_ produces a yellowish color. Concentrations of H_2_O_2_ were measured by a photometer at an absorbance of 400 nm. Calibration curves with known H_2_O_2_ concentrations (0–500 μM in PBS) were used for quantifications. No significant differences were found when the calibration curves were prepared in the presence of high nitrite/nitrate concentrations (both 2 mM) and low pH values (5.5 and 6.4).

### Quantification of TGF-β1

The levels of TGF-β1 in culture supernatants were determined on day 7 after treatment by using sandwich ELISA (DuoSet, R&D Systems, Abdingdon, UK) according to the manufacturer’s instructions.

### Quantitative immunocytochemical detection of gamma-H2AX

Within minutes of the induction of DNA double-strand breaks in somatic cells, histone H2AX becomes phosphorylated at serine 139 and forms γ-H2AX foci at the sites of damage [37]. One our after DBD/H_2_O_2_ treatments or UVB irradiation (500 mJ/cm^2^) as positive control, the cells were washed 3x with PBS and fixed with paraformaldehyde/PBS (4%; 30 min). After an additional fixation step with methanol (10 min; -20°C), the cells were washed 3x with PBS and permeabilized by 0.3% Triton X-100/PBS. After incubation with blocking buffer (5% BSA/PBS; 12 h; 4°C), the cells were stained with rabbit γ-H2AX -antibody (1:500; 60 min; 37°C; Abcam, Cambridge, UK), washed 3x with PBS and incubated for 60 min with an AlexaFluor488-conjugated donkey-anti-rabbit antibody (1:500; Invitrogen, Carlsbad, CA, USA) in blocking buffer. After washing steps (3x) and an incubation step with propidium iodide (1 μg/ml, 10 min; 37°C), the cells were visualized by using a fluorescence microscope (Axiovert, Zeiss, Wetzlar, Germany) and photos (2x) of each well were randomly taken. The number of nuclei and γ-H2AX foci were determined by using Fiji (National Institutes of Health, Bethesda, MD, USA).

### Statistical analysis

For statistical analysis, we used GraphPad Prism V 5.01 (San Diego, USA). Significant differences were evaluated by using repeated measures ANOVA followed by an appropriate post-hoc multiple comparison test (Tukey method). A p-value <0.05 was considered significant.

## Results

### DBD induces acidification and accumulation of nitrite, nitrite and H_2_O_2_

It is known that cold plasma can acidify media and increase the levels of nitrite, nitrate and perhaps H_2_O_2_. Therefore, to elucidate the impact of DBD treatment on cells, we first analyzed the pH and the concentrations of nitrite, nitrate and H_2_O_2_ in DBD-treated buffers (PBS). Our data revealed that under our experimental conditions, plasma treatment of PBS induced a significant increase in nitrite, nitrate and H_2_O_2_ concentrations coinciding with a decrease in pH (Fig 1B). For example, after a 300 s treatment of 250 μl PBS we found 1147.05 ± 452.78 μM of total nitric oxide derivatives (nitrate + nitrite). We calculated an accumulation rate of 1.00 ± 0.03 nmol/s. In parallel, the oxonium accumulation rate was 1.01 ± 0.20 nmol/s; thus, the pH value had dropped from 7.2 to 6.7 after 300 s plasma treatment. Coincidently, H_2_O_2_ and nitrite concentrations increased to 197.19 ± 24.25 μM and 294.09 ± 78.33 μM, respectively (Fig 1B)

### DBD treatment reduces the viability of fibroblasts

**Fig 2:**
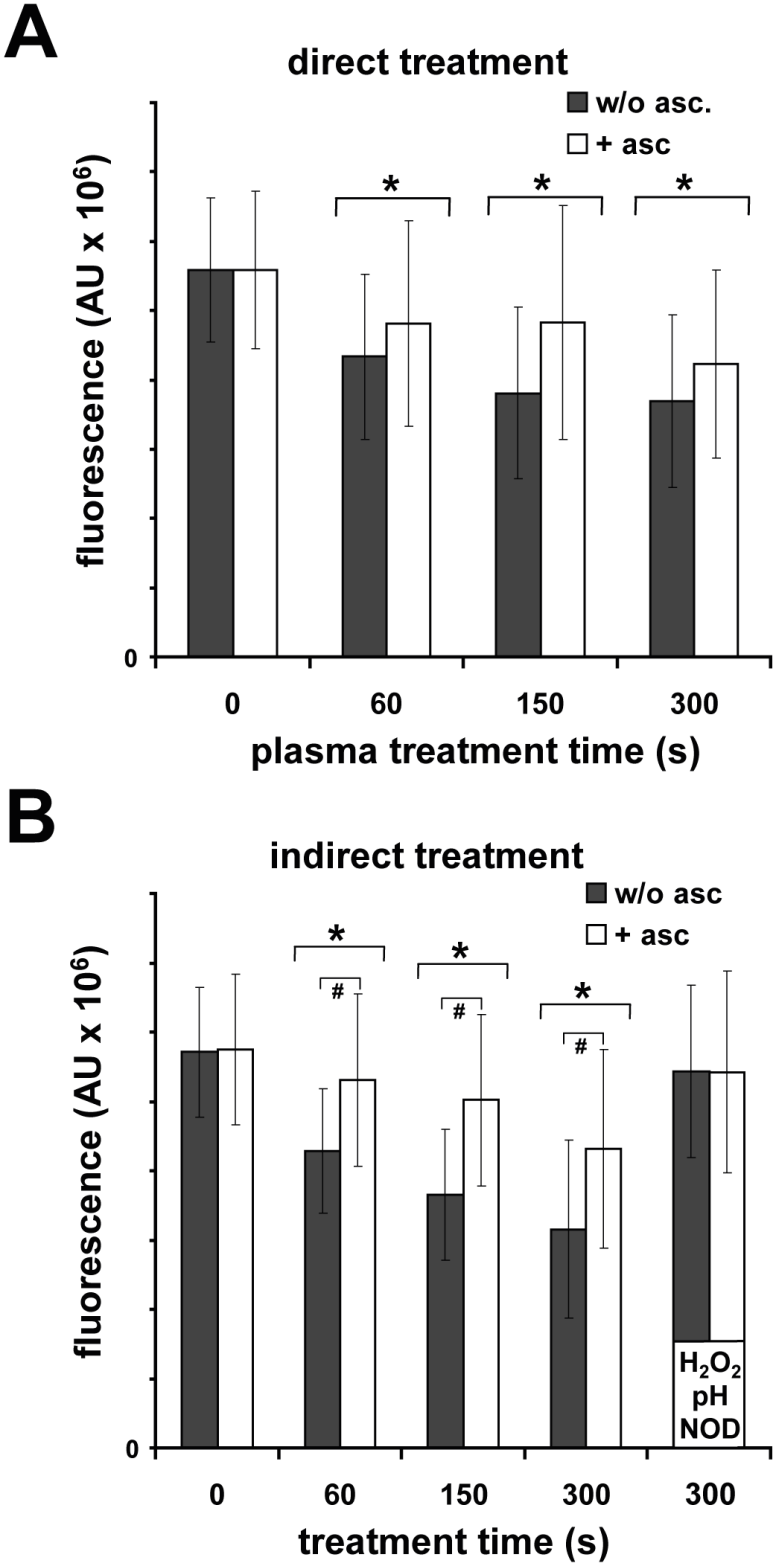
DBD treatment reduces fibroblast viability. **A** Human dermal fibroblasts were treated with DBD (0–300 s) ± ascorbate (**asc**; 1.0 mM) and cell viability was measured 24 h later by a resazurin-based assay. **B** Cells were exposed for 5 min to buffer ± ascorbate (1.0 mM), which had been DBD-treated (0–300 s) shortly before or exposed to a freshly prepared buffer with a pH of 6.7 (**pH**) containing hydrogen peroxide (200 μM; H2O2), nitrite and nitrate (**NOD**; 300 μM, 1.0 mM). Given are mean ± sd values (n = 5),*P < 0.05 as compared with the control values,#*P* < 0.05 as compared to respective values without ascorbate.

As many studies have reported plasma-induced toxicity in a number of cell types, we wanted to determine whether DBD treatment can influence the viability of human dermal fibroblasts. Preliminary experiments have shown that under our experimental conditions (Fig 1A), a minimal volume of 215 μl buffer (PBS) is necessary to avoid direct DBD-induced necrotic events. Using 250 μl, we could not observe either necrotic cells or cell detachment after treatment even after longer treatment times of up to 750 s. Analysis of cell viability 24 h after treatment revealed significant decreases (Fig 2A). For example, after direct DBD treatment for 300 s, the cell viability was ~66% in comparison to the untreated controls. The presence of the antioxidant sodium ascorbate could remarkably reverse (~75%) these observed effects, but not significantly. The plasma-induced accumulation of nitrite/nitrate/H_2_O_2_ in the media with coincident acidification suggests that the biological effects observed after DBD treatment, were not only mediated by the generation of short-living ROS. We therefore examined whether the buffer, which was DBD-treated separately from cells, has similar effects on the viability of fibroblasts. In addition, the buffer was acidified (pH 6.7) and nitrite, nitrate and H_2_O_2_, equivalent to amounts obtained after a 300 s DBD treatment, were freshly added to the cells and incubated for 5 min. Indeed, we observed a significant decrease in viability when the cells were exposed with DBD-treated buffer (e.g. ~55%, 300 s; Fig 2B). Here, the presence of ascorbate significantly reversed this effect (e.g. ~75%, 300 s). Incubation with acidified buffer containing nitrite/nitrate/H_2_O_2_ did not show any significant effect on cell viability.

### DBD treatment inhibits proliferation of fibroblasts

**Fig 3:**
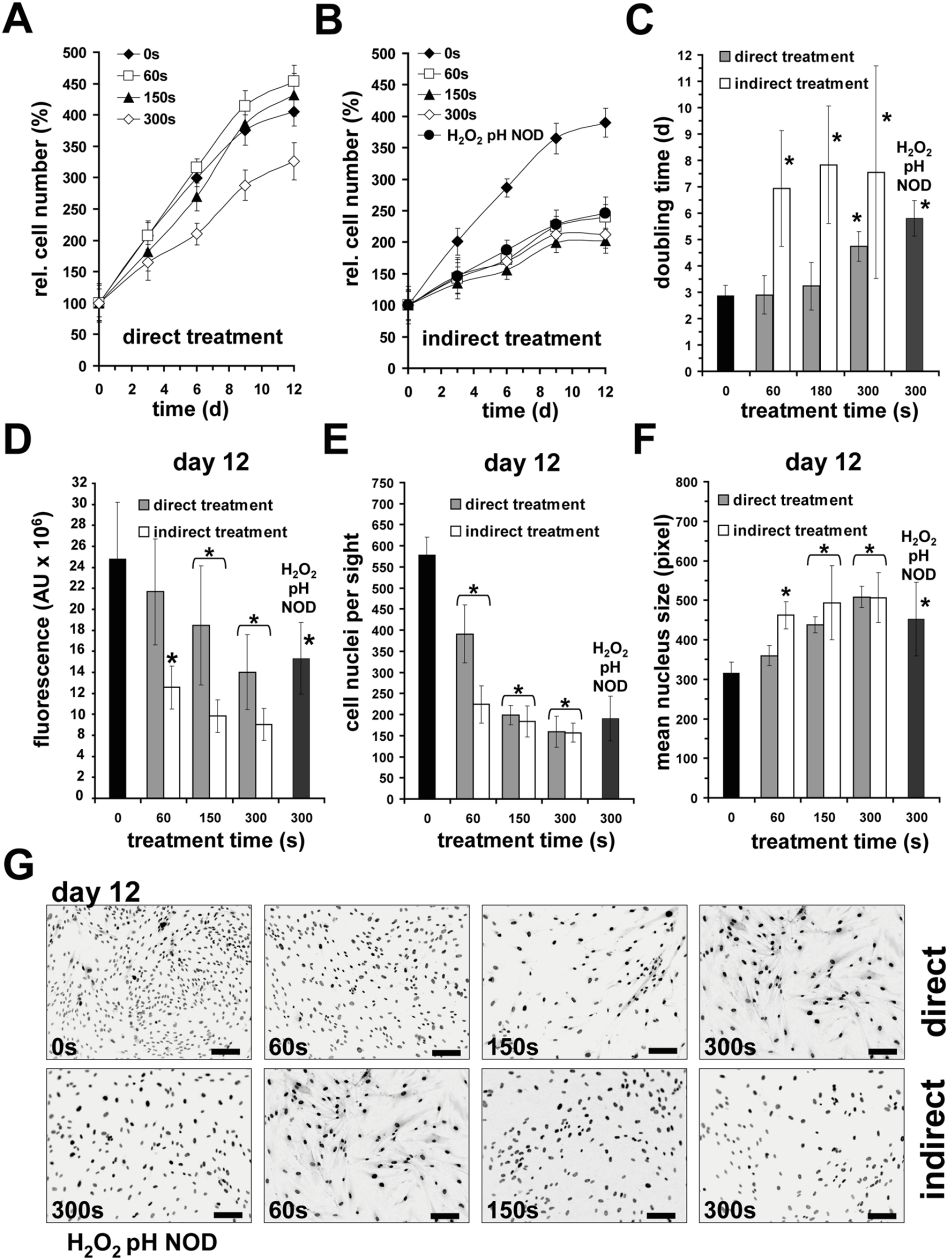
DBD treatment inhibits proliferation of human dermal fibroblasts. **A** Proliferation curve of remaining fibroblasts after direct DBD treatment measured by a resazurin-based assay. **B** Proliferation curve of remaining fibroblasts after exposure for 5 min to buffer, which was DBD-treated as indicated (indirect treatment). Additionally, fibroblasts were exposed to acidified buffer (**pH** 6.7) containing nitrite/nitrate (**NOD;** 300 μM / 1.0 mM) and **H**_**2**_ **O**_**2**_ (200 μM). **C** The calculated doubling times for proliferation curves A and B.**D** Values obtained by resazurin-based assay on day 12 after treatment. **E** Counted Hoechst 33342-stained nuclei on day 12 after treatment.**F** Quantification of mean fibroblast nucleus size on day 12 after treatment.**G** Representative microphotographs of Hoechst 33342-stained nuclei of fibroblasts on d12 after treatment. Bars = 200μm. Given are the mean ± sd values (n = 5),*P < 0.05 as compared with the control values.

The possible toxic effects of cold plasma were observed in many studies. However, few studies have focused on the proliferation of treated cells. To elucidate possible DBD-induced effects, we determined the proliferation rate of the remaining fibroblasts after direct DBD treatment or exposure to DBD-treated buffer. In addition, fibroblasts were incubated for 5 min with freshly prepared acidified buffer containing nitrite (300 μM), nitrate (1.0 mM) and H_2_O_2_ (200 μM) amounts equivalent to 300 s of DBD treatment. By using a resazurin-based assay, we observed that the proliferation rate was significantly lower after 300 s of direct DBD-treatment, whereas the rates were somewhat lower in shorter treatment times (Fig 3A). In contrast, the exposure of cells to DBD-treated buffer for 5 min results caused a significant breakdown of proliferation (Fig 3B). An analogous result was obtained when acidified nitrite/nitrate/H_2_O_2_ buffer were used (Fig 3B). The addition of ascorbate during treatment did not have any effect on proliferation (data not shown).

Further analysis revealed that the doubling time of directly DBD-treated cells was significantly prolonged after a 300 s treatment in comparison to the untreated control (4.8 ± 0.6 d vs. 2.8 ± 0.4d; Fig 3C). Incubation of fibroblasts for 5 min with DBD-treated buffer (60–300 s) led to doubling times of 6–8 d. Also the incubation of fibroblasts with acidified buffer containing nitrite/nitrate/H_2_O_2_ significantly prolonged doubling time (5.8 ± 0.7 d). Since the resazurin-based assay only gives a measure of metabolic active cells, we therefore additionally validated the cell number by manual counting of Hoechst-stained nuclei on day 12 after treatment. Direct and indirect DBD treatment (60–300 s) as well as incubation of cells with acidified nitrite/nitrate/H_2_O_2_ buffer led to a significant decrease in cell number (Fig 3E and 3G). Here, 150–200 cell nuclei were counted for treatments longer than 60 s, whereas we found three-fold more nuclei (578 ± 42) in the untreated control.

Interestingly, comparing the absolute fluorescence signals obtained from the resazurin assay (Fig 3D) to the number of cell nuclei (Fig 3E) showed some discrepancy on day 12. Here, using resazurin, the values of direct treatments were reduced but not to such an extent as the number of cell nuclei. For example, a direct DBD-treatment (150 s) led to a strong reduction in the number of nuclei (-66%), whereas the resazurin-based assay showed a moderate reduction (-28%), suggesting that not only the number of active cells but also the metabolism and/or phenotype of fibroblasts may be influenced by DBD-treatment. Indeed, an analysis of the mean size of nuclei revealed that the cell nuclei were roughly 1.6-fold larger on 12 d after direct and indirect DBD treatment, suggesting a DBD-induced cell cycle arrest or reduced mitotic activity. As the exposure of fibroblasts to acidified buffer containing nitrite/nitrate/ H_2_O_2_ showed similar effects, we therefore subsequently examined which of the components may inhibit cell proliferation.

**Fig 4:**
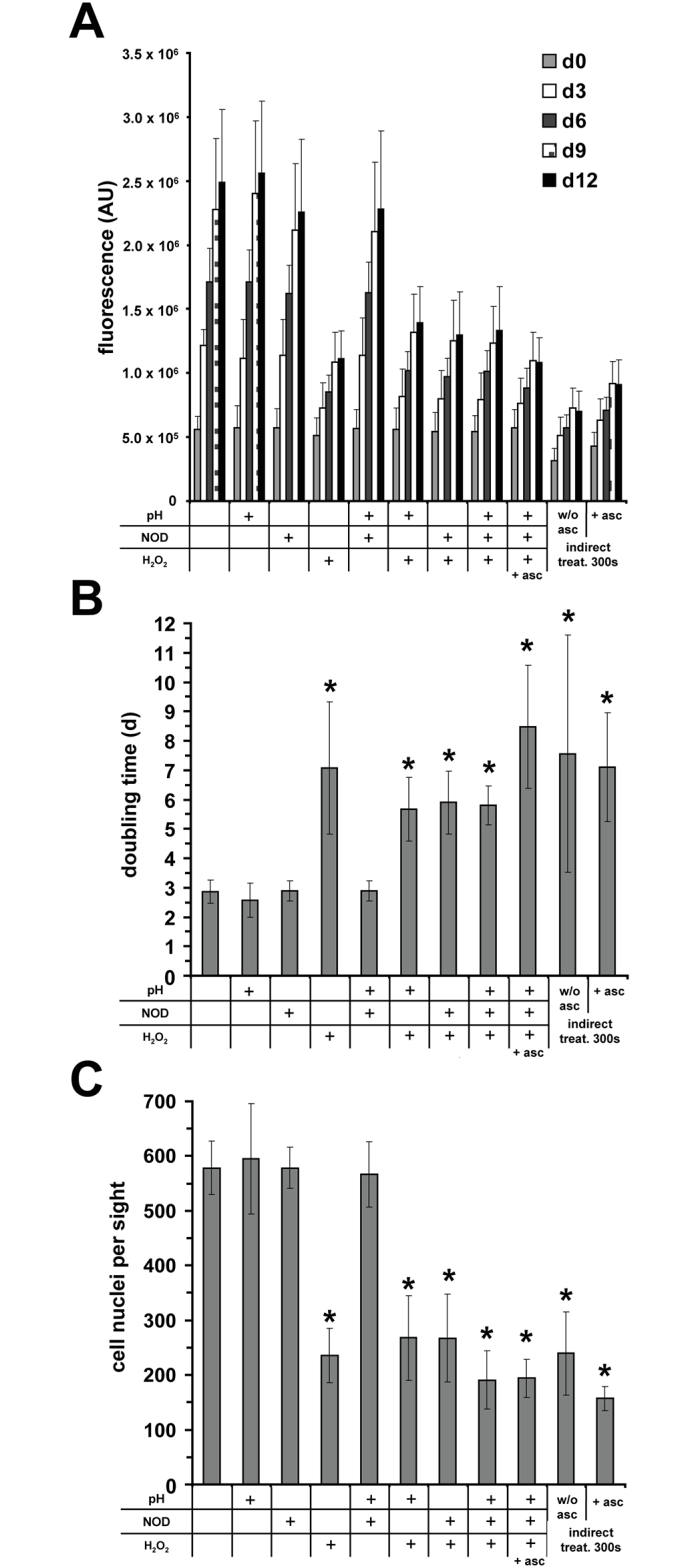
Hydrogen peroxide reduces proliferation of fibroblasts. Fibroblasts were exposed (5 min) to various combinations of hydrogen peroxide (H_2_O_2_; 200 μM), nitrite/nitrate (NOD; 300 μM/1.0 mM) and acidified PBS (pH 6.7) or normal PBS (pH 7.2) and sodium ascorbate (**asc**; 1 mM).The used concentrations were equivalent to concentrations found after 300 s DBD treatment. Additionally, fibroblasts were exposed for 5 min to DBD-treated PBS (300 s) with or without sodium ascorbate. **A** shows fluorescence values obtained by a resazurin-based assays as the measure for cell viability after treatment as indicated. **B** Calculated doubling time using obtained resazurin values. **C** Number of Hoechst 33342-stained nuclei on day 12 after treatment. Given are the mean ± sd values (n = 5),*P < 0.05 as compared with the control values.

Using various combinations, we found that the presence of H_2_O_2_ was necessary to significantly inhibit proliferation, to reduce the number of cell nuclei and to prolong doubling time to the same extent as an indirect DBD treatment (300 s), whereas acidification and nitrite/nitrate alone or combined did not reveal any effects (Fig 4A–4C). Furthermore, after direct and indirect DBD treatment (0–300 s) as well as exposure to H_2_O_2_ (0–1 mM, 300 s) no significant increase in gamma-H2AX foci as early marker of DNA double-strand breaks [37] could be found. However, an increasing tendency of gamma-H2AX correlating with treatment time could be recognized, particularly after direct treatments (S1 Fig).

### DBD treatment affects fibro-/myofibroblast differentiation

**Fig 5:**
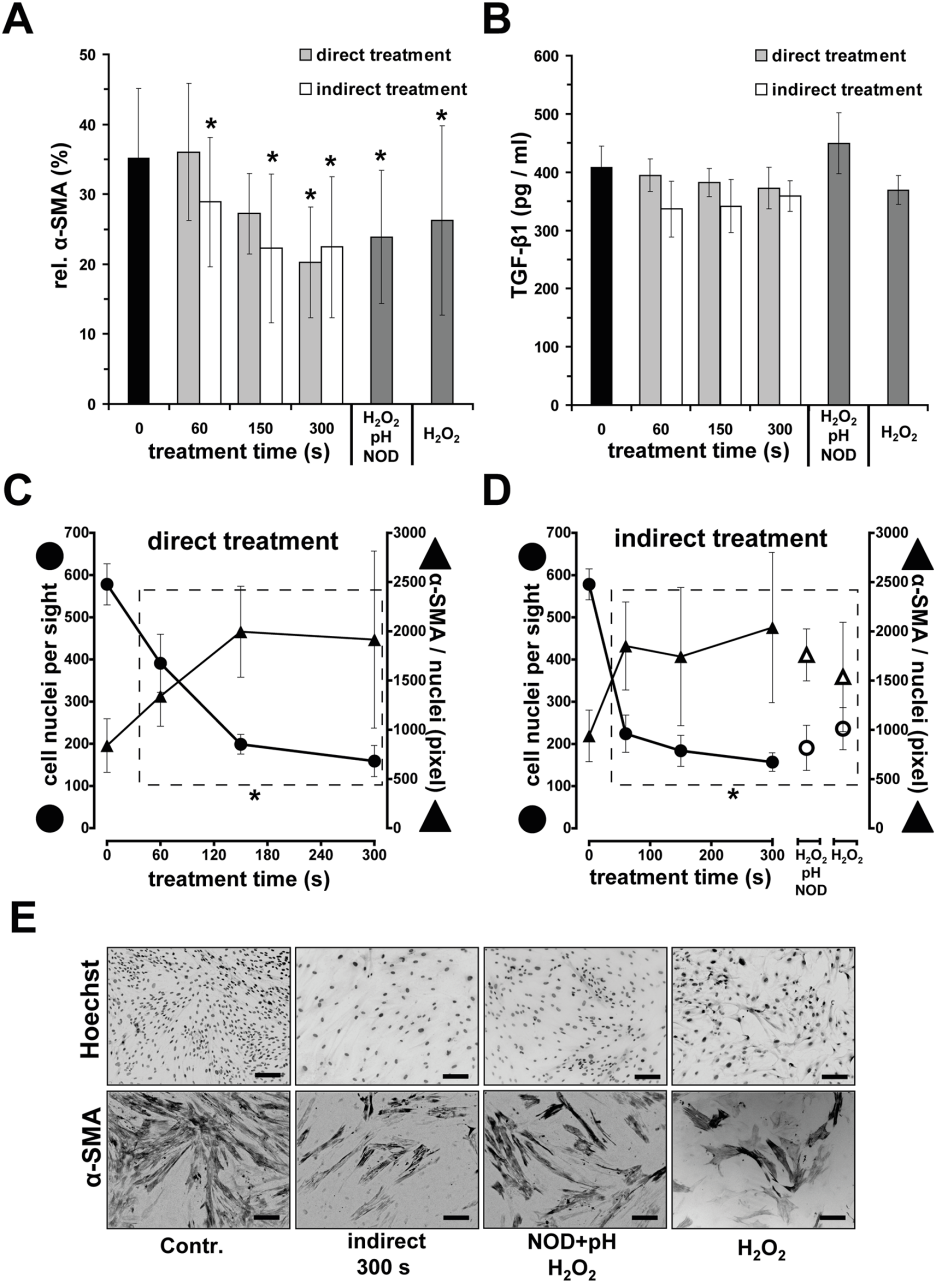
DBD treatment interferes with fibroblast/myofibroblast differentiation. **A** Quantification of alpha-smooth muscle actin of fibroblasts (α-SMA) 12 days after direct/indirect DBD treatment. **B** TGF-β1 concentrations in the supernatants seven days after treatment. **C** and **D** show number of Hoechst 33342 stained cell nuclei and α-SMA positive-stained area normalized to number of nuclei in 1.4 mm² cell culture plate 12 days after DBD treatment. **E** Representative microphotographs of Hoechst 33342-/α-SMA-stained fibroblasts.In control experiments, fibroblasts were exposed (5 min) to acidified PBS (pH 6.7) containing concentrations of nitrite/nitrate (**NOD**; 300 μM/1.0 mM) and hydrogen peroxide (**H2O2**; 200 μM), which are equivalent to concentrations obtained after a 300 s DBD treatment. Additionally, fibroblasts were exposed to PBS (pH 7.2) containing only H_2_O_2_ (200 μM). Given are the mean ± sd values (n = 5),*P < 0.05 as compared with the control values.

Myofibroblast activity during wound healing is crucial and an inhibition of differentiation of fibroblast to myofibroblast may result in problems in wound closure and healing. Thus, we evaluated the amount of myofibroblasts 12 days after DBD treatment by visualization of the myofibroblast marker α-smooth muscle actin (α-SMA). We observed that the α-SMA^+^ signals were significantly reduced by direct and indirect DBD treatments as compared to the untreated control. Particularly, after longer treatments times (300 s), only 20.2 ± 7.9% (direct) or 22.5 ± 10.1% (indirect) of the cell culture bottom area was positively stained, whereas the control showed 35.2 ± 9.9%. Analogous results could be achieved by the exposure of cells to H_2_O_2_ (Fig 5A). However, TGF-β1 levels were not significantly changed (Fig 5B).

With regard to the number of cells being coincidently reduced by DBD treatment, we noted a significant increase in α-SMA^+^ signal normalized to cell number by direct or indirect DBD treatment (Fig 5C and 5D).

## Discussion

Since non-thermal plasma has many potential clinical applications, for example, in the treatment of contaminated wounds or impaired wound healing, this study has addressed the interaction of plasma with dermal fibroblasts, which play an important role in wound healing.

Many studies have demonstrated that non-thermal plasma can produce dose-dependent effects ranging from increased cell proliferation to cell death [38–44]. It is hypothesized that cell death in response to non-thermal plasma is primarily through the induction of apoptosis as an effect of the production of reactive oxygen/nitrogen species, which can be scavenged by antioxidants [21].

It has also been demonstrated that *in situ* generation of the hydroxyl radical from superoxide anion and hydrogen peroxide (H_2_O_2_) plays a critical role in plasma-induced cell death [45].

Using DBD as CAP source, we have observed similar effects on fibroblast viability already after clinically relevant treatment times [46]. Here, the antioxidant ascorbate could attenuate toxic effects. Determining the toxic range in preliminary experiments, we could not find any toxic effects in human foreskin fibroblasts after 15 and 30 s direct DBD treatments (data not shown). However, the exposure of fibroblasts to DBD-treated buffer also reduced cell viability, suggesting that besides high-reactive, short-living radicals (superoxide, singlet oxygen, hydroxyl radical) are also a more stable reactive species such as H_2_O_2_ within the buffer or other plasma-induced chemical and physical changes (e.g. accumulation of nitrite/nitrate, pH, osmolarity) may be responsible for the observed effects.

Mimicking indirect DBD treatment (300 s) by the acidification of buffer and addition of equivalent amounts of nitrite/nitrate/H_2_O_2_ has shown slight but not significant effects on viability. In contrast, an indirect DBD treatment reduced cell viability by more than 50%, thereby suggesting that the final products such as H_2_O_2_, nitrite and nitrate were not the main responsible agents for toxicity. Furthermore, we observed significant inhibition of fibroblast proliferation and nuclei swelling, which was more pronounced by indirect DBD treatments. In preliminary experiments with foreskin fibroblasts, we could not find any effects on proliferation after 15 and 30 s direct DBD treatments (data not shown). Although we observed no effects on viability, acidified buffer containing nitrite/nitrate/H_2_O_2_ could significantly inhibit cell proliferation to the same degree as indirect treatments. Further experiments revealed that these inhibition effects were solely dependent on the presence of H_2_O_2_ and not on nitrite/nitrate and/or acidification. It is well-known that brief exposures to H_2_O_2_ (50 μM; 1 h) can induce delayed cell death and a prolonged inhibition of cell division in endothelial cells[47]. Sub-lethal treatment of H_2_O_2_ (200 μM; 2 h) “stunned” also fibroblast F65 cells and caused them to enter a state resembling senescence [48].

In addition, H_2_O_2_ can cause DNA damage, which in turn induces cell toxicity and inhibition of proliferation [49]. In our experiments we could not find a significant increase of γ-H2AX as a marker for DNA double-strand breaks after direct or indirect plasma treatment or H_2_O_2_

However, a treatment time-dependent, but not significant, increase in γ-H2AX levels could be observed after direct DBD treatment. It is important to mention that in contrast to most studies, the cells in our experiments were only exposed for a short time (300 s) to a comparably low concentration of H_2_O_2_; thus, it seems probable that H_2_O_2_ -induced DNA damages can occur after longer DBD treatments. In general, the biological impact of H_2_O_2_ depends on cell type, cell cycle status, cellular antioxidative defense, concentration, exposure time and also the cell environment. Therefore, toxic or antiproliferative doses of H_2_O_2_ treatment can vary dramatically between studies. The same applies for cold plasma treatments; however, here the use of different plasma sources, operation parameters and therefore many more factors (e.g. radicals, UV, temperature, electric fields etc.) further complicate the matter.

Thus, it is not surprising that, in contrast to other studies [24,50], our data indicate that toxic effects by DBD are not predominantly mediated by H_2_O_2_.

In our experiments we used human foreskin fibroblasts, which in contrast to adult skin fibroblasts undergo myofibroblast differentiation in cell culture media without addition of inductors such as TGF-β1. Apart from proliferation, the differentiation of fibroblasts to myofibroblasts was affected by DBD treatment (direct/indirect) as well as by H_2_O_2_-containing buffers. On the one hand, the number of α-SMA^+^ cells was decreased by treatment, and on the other hand, normalized to cell nuclei, the differentiation frequency was increased. In addition, TGF-β1 levels did not change significantly.

We hypothesize that reactive species such as hydroxyl/superoxide radicals and nitrogen dioxide, which can be scavenged by the antioxidant ascorbate, are partly responsible for loss of cell viability. In addition, we assume that DBD-generated nitrite/nitrate and acidic pH under certain conditions are also involved in toxicity as observed previously in adult skin fibroblasts. However, further unknown mechanisms may take part in the meditation of cell toxicity. Our results indicate that myofibroblast differentiation can be modified by DBD treatment and H_2_O_2_. In this context, one has to mention again that plasma-mediated effects differ between cell types and cell states. Resting, slow proliferating and/or predifferentiated cells such as proto-myofibroblasts producing TGF-β1 [51], may be less sensitive to DBD/ROS exposure and preferably remain so after treatment. Additionally, generated H_2_O_2_ inhibits proliferation of the remaining cells. These combined effects could explain that on the one hand, DBD treatment reduces the total number of myofibroblasts, but on the other hand, increases the relative number of myofibroblasts. It is also possible that DBD treatment induces differentiation in fibroblast by further mechanisms, for example, intracellular oxidative stress and/or cell cycle arrest, which were described to promote differentiation processes [52,53].

In conclusion, understanding the mechanisms underlying the different effects of CAP on cell viability, proliferation and differentiation is essential for clinical use. We demonstrated that direct and indirect DBD treatments can reduce fibroblast viability *in vitro*. This reduction is not dominantly mediated by generated H_2_O_2_, which in turn seems to be responsible for the observed proliferation, inhibition and reduction of myofibroblasts. Due to antibacterial effects, cold plasma treatment represents an effective weapon against resistant bacteria in contaminated wounds. In a recent clinical trial, Brehmer *et al*. have shown the reduction of the bacterial load of chronic wounds by a DBD device (PlasmaDerm; 45 s/cm^2^) without harmful side effects [46]. Moreover, by acidification, by generating various biological-active reactive species and by enhancing the local microcirculation cold plasma may positively affect cell proliferation and healing process, particularly in chronic wounds [28]. In addition, a CAP-induced inhibition of cell proliferation in the context of hyperproliferative skin diseases, such as excessive scarring or psoriasis, may have beneficial therapeutic effects. However, “Right Drug, Right Dose, Right Time” as one basic pharmacological principle also needs to be applied in this matter to avoid disadvantageous effects—for example, delayed wound healing. Therefore, further investigations and clinical trials are necessary to evaluate the possible applications of CAP in clinical use.

## Supporting Information

S1 Fig DBD treatment does not induce significantly DNA double-strand breaks. **A** Immunocytochemical quantification of gamma-H2AX histone in fibroblasts as marker for DNA double-strand breaks one hour after direct/indirect DBD treatment and **B** 5 min exposure to hydrogen peroxide (0–1000 μM). **C** Representative microphotographs of propidium iodide (**PI**) and gamma-H2AX histone stained fibroblasts treated as indicated. Cells were treated in the presence of ascorbate (**asc**; 1 mM) or irradiated with **UVB** (500 mJ/cm^2^) as positive control. Given are the mean ± sd values (n = 5), *P < 0.05 as compared with the control values.
(TIF)
