# Supplementary figures and images for: Non-Thermal Dielectric Barrier Discharge (DBD) Effects on Proliferation and Differentiation of Human Fibroblasts Are Primary Mediated by Hydrogen Peroxide
Source: PLoS One. 2015 Dec 14;10(12):e0144968. doi: 10.1371/journal.pone.0144968 (PMC4682795; doi:10.1371/journal.pone.0144968)

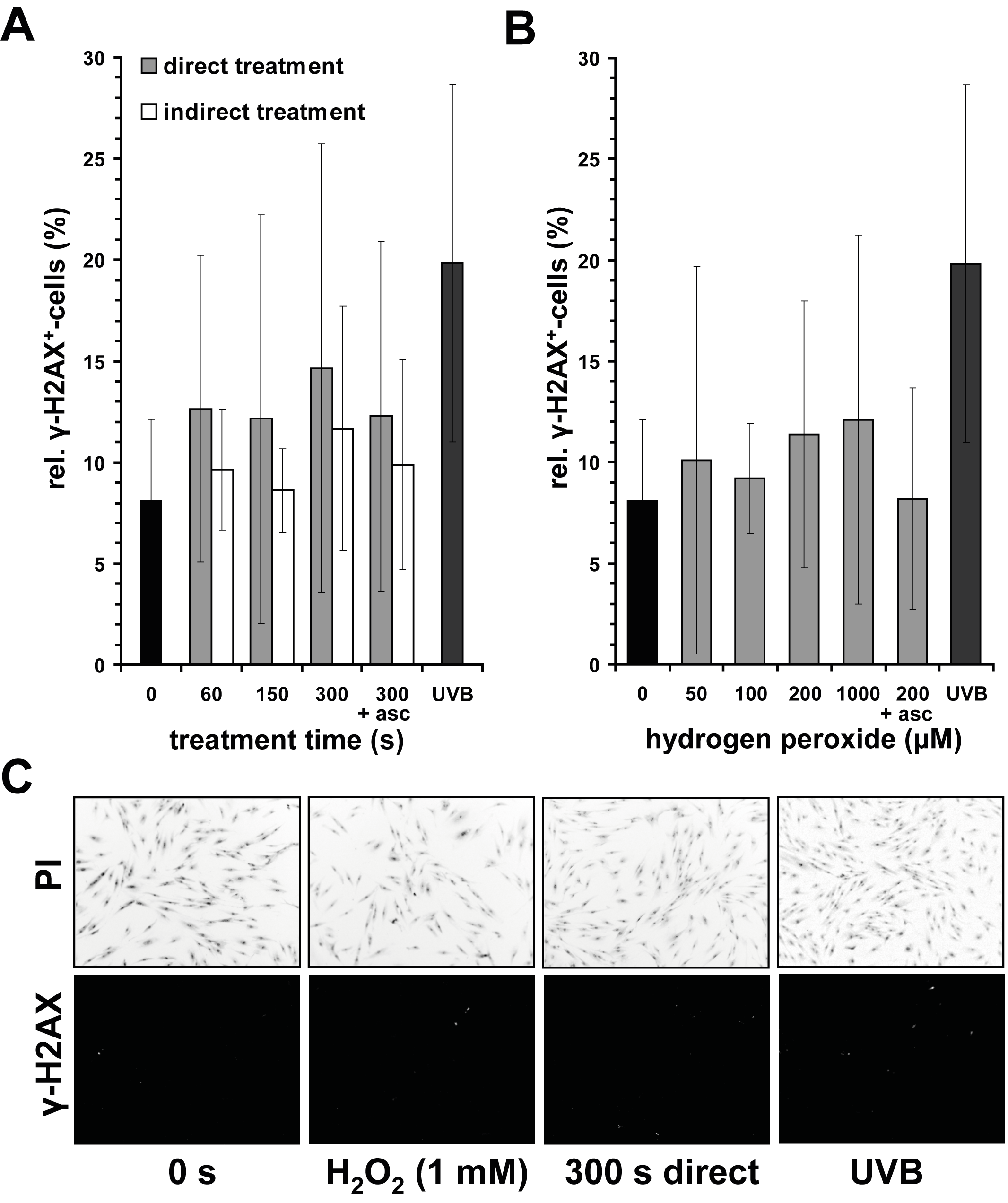

Supplement: S1 Fig — A Immunocytochemical quantification of gamma-H2AX histone in fibroblasts as marker for DNA double-strand breaks one hour after direct/indirect DBD treatment and B 5 min exposure to hydrogen peroxide (0–1000 μM). C Representative microphotographs of propidium iodide (PI) and gamma-H2AX histone stained fibroblasts treated as indicated. Cells were treated in the presence of ascorbate (asc; 1 mM) or irradiated with UVB (500 mJ/cm2) as positive control. Given are the mean ± sd values (n = 5), *P < 0.05 as compared with the control values. (TIF) [file pone.0144968.s001.tif]
